# Unlocking the potential of dimethyl fumarate: enhancing oncolytic HSV-1 efficacy for wider cancer applications

**DOI:** 10.3389/fimmu.2023.1332929

**Published:** 2023-12-19

**Authors:** Akram Alwithenani, Zaid Taha, Max Thomson, Andrew Chen, Boaz Wong, Rozanne Arulanandam, Jean-Simon Diallo

**Affiliations:** ^1^ Centre for Cancer Therapeutics, Ottawa Hospital Research Institute, Ottawa, ON, Canada; ^2^ Department of Biochemistry, Microbiology, and Immunology, Faculty of Medicine, University of Ottawa, ON, Canada; ^3^ Department of Clinical Laboratory Science, Faculty of Applied Medical Science, Umm Al-Qura University, Makkah, Saudi Arabia

**Keywords:** HSV-1, DMF, cancer, cancer therapeutics, human specimens, fumarate esters

## Abstract

Immunotherapy and specifically oncolytic virotherapy has emerged as a promising option for cancer patients, with oncolytic herpes simplex virus-1 (oHSV-1) expressing granulocyte macrophage colony stimulating factor being the first OV to be approved by the FDA for treatment of melanoma. However, not all cancers are sensitive and responsive to oncolytic viruses (OVs). Our group has demonstrated that fumaric and maleic acid esters (FMAEs) are very effective in sensitizing cancer cells to OV infection. Of note, these FMAEs include dimethyl fumarate (DMF, also known as Tecfidera^®^), an approved treatment for multiple sclerosis and psoriasis. This study aimed to assess the efficacy of DMF in combination with oncolytic HSV-1 in preclinical cancer models. We demonstrate herewith that pre-treatment with DMF or other FMAEs leads to a significant increase in viral growth of oHSV-1 in several cancer cell lines, including melanoma, while decreasing cell viability. Additionally, DMF was able to enhance *ex vivo* oHSV-1 infection of mouse-derived tumor cores as well as human patient tumor samples but not normal tissue. We further reveal that the increased viral spread and oncolysis of the combination therapy occurs via inhibition of type I IFN production and response. Finally, we demonstrate that DMF in combination with oHSV-1 can improve therapeutic outcomes in aggressive syngeneic murine cancer models. In sum, this study demonstrates the synergistic potential of two approved therapies for clinical evaluation in cancer patients.

## Introduction

Oncolytic virus (OV) therapy is a promising new option for cancer treatment. OVs represent a class of therapeutically useful viruses that preferentially infect and kill cancer cells while leaving normal cells relatively unharmed (1, 2). This tumor tropism is derived from the aberration of anti-viral responses commonly found within tumor cells. Enhanced virus growth and reduced viral clearance is typically observed in innate immunity-compromised, transformed cells (3).

Several kinds of OVs have been described with the potential to eradicate tumors, leading to long term anti-tumor immunity. In the last decade, oncolytic Herpes Simplex Virus (oHSV-1) has made its way to the clinic to treat advanced melanoma (unresectable stage IIIB to IV) (4). The first FDA-approved OV called T-VEC or Imlygic^®^ is based on an engineered version of HSV-1 devoid of γ34.5. In June 2021 another genetically engineered OV based on HSV-1 (G47Δ), DELYTACT^®^ received conditional and time-limited approval for the treatment of malignant gliomas in Japan (5). Both HSVG47Δ and HSV.γ34.5 have the ICP34.5 gene deleted to provide more selective tumor replication (6). These strains have been shown to induce tumor regression and prolong survival significantly in different animal models of cancers such as glioma, melanoma and ovarian cancer (7, 8). Furthermore, it has been shown that HSV-1 with mutations (HSV.n212) or deletions (HSV.d810) in the immediate early gene ICP0, which encodes a protein responsible for overcoming aspects of the host IFN response (suppressing IRF-3 and IRF-7), has oncolytic properties (9).

Unfortunately, not all cancers are sensitive and responsive to oncolytic virotherapy. Numerous studies have shown that a subset of cancer patients are resistant to OV treatment due to various mechanisms, including a functional type I interferon pathway and an immunosuppressive tumor microenvironment (TME) (10). Several strategies to overcome the resistance and maximize the therapeutic efficacy of OV treatment have been under investigation (11, 12). One promising strategy to increase the impact of the OV is to administer pharmacological drugs that facilitate OV infection of cancer cells but not normal cells. Multiple promising drug classes have been discovered to this end (13–16). These drugs can transiently decrease the type I IFN response to allow OVs to gain a foothold and propagate within the tumor (17).

One of the drugs previously described to promote effectiveness of oncolytic virotherapy is Dimethyl Fumarate (DMF). DMF is an approved drug for psoriasis taken as an oral therapy, and since 2013 DMF has also been approved for relapsing multiple sclerosis (18). In the context of cancer treatment, DMF has demonstrated anti-tumor properties by reducing tumor growth and metastasis (19, 20). Specifically, DMF was shown to suppress the nuclear factor kappa B (NFκB) pathway and induce oxidative stress through cellular Reactive oxygen species (ROS); resulting in tumor regression (21). DMF has also been reported to suppress cell proliferation in multiple breast cancer cell lines via inhibition of NFκB activity (22). In addition, DMF was shown to sensitize tumors to chemotherapy (23), as NFκB regulates several genes that are involved in chemotherapy resistance. In a previous study published by our group (24), it was shown that DMF effectively enhanced the spread and oncolysis of Vesicular Stomatitis Virus (VSVΔ51) across a range of resistant cancer cell lines, including human clinical specimens. The combination of DMF and VSVΔ51 demonstrated significant efficacy in various immune-competent cancer model such as murine colon carcinoma (CT26.wt). Notably, DMF’s capacity to augment viral spread can be attributed to its capability in suppressing type I interferon (IFN).

Drawing on DMF’s established effectiveness in cancer therapy, its clinical availability and its successful combination with oncolytic VSVΔ51 (24), as well as the clinical progress of oncolytic HSV, the amalgamation of these modalities emerges as a compelling therapeutic strategy. DMF’s demonstrated potential to suppress tumor growth, combined with oncolytic HSV’s tumor-selective replication and cytotoxicity, presents an opportunity for a multifaceted approach with enhanced therapeutic impact. The objective of this study is to demonstrate the preclinical feasibility of employing DMF in conjunction with oncolytic HSV-1, both *in vitro* and *in vivo* settings. In the current study, our investigation exploited ICP0 null HSV-1 strains, denoted as HSV.n212 and HSVd810, alongside ICP34.5-deficient HSV-1 strains, specifically HSV.γ34.5 and HSVG47Δ.

## Materials and methods

### Cell lines

OHRI-MEL-13 (primary human melanoma cells) was obtained as a generous gift from Dr. Carolina Ilkow of the Ottawa Hospital Research Institute (Ottawa, Canada) (25).

All other cell lines used in this study including 786-0 (human renal cell adenocarcinoma), HT29 (human colon carcinoma), 4T1 (mouse breast carcinoma), CT26.wt (mouse colon carcinoma), S-180 (mouse sarcoma), CT2A (mouse glioma), Vero (African Green Monkey Kidney, CCL81). Cells were obtained from the American Type Culture Collection (ATCC) and maintained in Dulbecco’s modified Eagle’s medium (DMEM, HyClone, Waltham, Massachusetts, or Corning, Manassas, Virginia), supplemented with 1% penicillin-streptomycin (Gibco) and 10% fetal bovine serum (FBS; VWR, Mississauga, Ontario).

All cells were incubated at 37°C in a 5% CO2 humidified incubator and routinely tested for mycoplasma contamination by Hoechst staining and PCR (Diamed, Mississauga, Ontario, Catalog # ABMG238).

### Viruses, purification, and quantification

ICP0-Null HSV including (HSV.n212) expressing GFP and (HSV.d810) expressing GFP was obtained as a generous gift from Dr. Karen Mossman of McMaster University (Hamilton, Canada). HSV titers were determined by a standard plaque assay on Vero cells according to a previously published protocol (14). Herpes simplex virus gamma 47 delta (HSV-G47Δ) has deletions in the g34.5 and a47 genes, and the inactivating insertion of LacZ into ICP6 was obtained as a generous gift from Dr. Samuel Rabkin of Harvard University. HSV titers were determined by a standard plaque assay on Vero cells according to a previously published protocol (13). The oncolytic HSV.n212 and HSVG47Δ were grown and tittered on Vero cells. Briefly, HSV was added at multiplicity of infection (MOI) of 0.05 to 95% confluent Vero cells in roller bottles in a total volume of 25 ml of complete DMEM. Infected Vero cells were incubated at 37°C with 5% CO2 for 48-72 h or when reach ~90% CPE (cell syncytia) was observed. Supernatants and cells were collected. Supernatants were pelleted at 25,000 RPM for 2 hours. Cells were frozen and thawed twice, then pelleted at 1200 RPM for 10 minutes to clear cell debris. The virus contained within the cleared supernatant was combined with the pellets of the first supernatant and purified using a 36% sucrose gradient.

### Plaque assay

Vero cells were used to titer HSV infected samples. Briefly, all infectious samples were serially diluted then transferred into monolayer of Vero cells. After an incubation of 60 mins, an overlay of CMC: DMEM were added for 72 hours. For visualization of plaques, a 0.5% Crystal Violet solution were added to the wells.

### Viral growth curves

786-0 were cultured overnight to reach in a confluency next day. Subsequently, the cells were infected with herpes simplex virus (HSV) at two different MOI: 0.01, utilized for multi-step growth curve analysis, and 1.0, for single-step growth curve analysis. MOI 1.0-infected cells underwent a 60-minute incubation, followed by a washing step and media replenishment. The cells were then maintained for up to 72 hours post-infection (hpi), with 200 µl of supernatant collected and stored at -80°C at specific time intervals: 0, 4, 8, 12, 24, 32, and 48 hpi. Viral titers in the collected samples were subsequently quantified using plaque assays, following established procedures.

### Drugs

Dimethyl fumarate (DMF) (Sigma-Aldrich) was resuspended in 100% DMSO to 100mM and further diluted at indicated dilutions before use in all *in-vitro* experiments. For *in-vivo* experiments, DMF was dissolved in 100% DMSO or 0.8% methyl cellulose at 50mg/mL diluted at indicated dilutions before use.

Monomethyl fumarate (MMF), Diethyl fumarate (DEF), Dimethyl maleate (DMM), Diethyl maleate (DEM) and Fumaric acid (FA) were all obtained from (Sigma-Aldrich) and all resuspended in 100% DMSO to 100mM and further diluted at indicated dilutions before use in all *in-vitro* experiments.

### Cell viability assay

The assessment of cellular metabolic activity was conducted through the use of Resazurin (metabolic dye) (Millipore Sigma, cat. SI03200) according to the manufacturer’s protocol. 10% of resazurin were added to all samples for 1-2 hours, depending on the cell line. Using the BioTek Microplate Reader (BioTek, Winooski, VT, USA) and Gen5 2.07 software. Fluorescence was measured at 590 nm upon excitation at 530 nm. Readings were expressed relative to the average of the uninfected, mock treated condition.

### IFN-β ELISA

786-0 cells were first treated with DMF at 150uM for a duration of 4 hours then infected with HSV at an MOI of 0.1. The 786-0 cell supernatant, obtained 24 hours post-infection following treatment and infection, underwent assessment for the concentration of human IFN-β. This quantification was conducted using the Human IFN Beta ELISA Kit (PBL Assay Science, cat. 41410), adhering to the guidelines provided by the manufacturer. Absorbance readings were taken using BioTek Cytation C10 Confocal Imaging Reader.

### Quantitative real-time PCR

786-0 cells were first treated and then infected as described. After 24 hours, the RNA from the lysed cells was homogenized with the QIAshredder (Qiagen, cat. 79656) and extracted with the QIAGEN Rneasy kit (Qiagen, cat. 74106) following the manufacturer’s protocol. The RNA was quantified using a NanoDropTM One Microvolume UV-Vis Spectrophotometer (Thermo Fisher Scientific, Rockford, IL). RevertAid First Strand cDNA Synthesis Kit was used to convert 1 ug of RNA to cDNA. The real-time PCRs were carried out on a 7500 Fast Real-Time PCR system (Applied Biosystems) using the Applied Biosystems PowerUp SYBR Green Master Mix (ThermoFisher Scientific) following the manufacturer’s protocol. The Pfaffl method was used to calculate gene expression.

### *In vivo* mouse tumor models

All experiments were conducted following the University of Ottawa Animal Care and Veterinary Service guidelines for animal care under protocols OHRI-2264 and OHRI-2265.

#### Dose escalation study

BALB/c mice that were 6 weeks old and obtained from Charles River Laboratories were implanted subcutaneously with syngeneic CT26.wt colon carcinoma cells in the right flank using 100μL PBS. Once the tumors reached a volume of approximately 100 mm^3^ after 11 days, the mice received injections of HSV (1 x 10^8^ pfu) directly intratumorally, either once, twice, thrice, or six times. Tumor size was monitored every other day using an electronic caliper, and their volumes were calculated using the formula (length × width^2^)/2. The mice were euthanized when tumor volumes exceeded 1500 mm^3.^

#### Route of administration study

BALB/c mice that were 6-8 weeks old and obtained from Charles River Laboratories were implanted subcutaneously with syngeneic CT26.wt colon carcinoma cells in the right flank using 100μL PBS. Once the tumors reached a volume of approximately 100mm^3^ after 11 days, mice were injected either intratumorally or given DMF by oral gavage (200mg/kg). Five hours later, a bolus of 25μL PBS containing 1 x 10^8^ pfu of HSV.n212 was injected intratumorally. This treatment was repeated two more times, with a one-day interval between each treatment.

#### CT26.wt model

BALB/c mice that were 6-8 weeks old and obtained from Charles River Laboratories were implanted subcutaneously with syngeneic CT26.wt colon carcinoma cells in the right flank using 100μL PBS. Once the tumors reached a volume of approximately 100mm^3^ after 11 days, mice were injected intratumorally with either DMF (200mg/kg) or vehicle alone. Five hours later, a bolus of 25μL PBS containing 1 x 10^8^ pfu of HSV.n212, or PBS alone, was injected intratumorally. This treatment was repeated two more times, with a one-day interval between each treatment.

#### 4T1 model

BALB/c mice that were 6 weeks old and obtained from Charles River Laboratories were implanted subcutaneously with 5 x 10^5^ 4T1 syngeneic 4T1 mammary carcinoma cells in 100μL PBS in their right flanks. After 9 days, when the tumor volumes reached about 100mm^3^, DMF (200mg/kg) or vehicle alone was injected intratumorally. Five hours later, a bolus of 25μL PBS containing 1 x 10^8^ pfu of HSV.n212, or PBS alone, was injected intratumorally. This treatment was repeated two more times, one day apart.

Mice were randomized to different treatment groups based on tumor size prior to the first treatment. For survival studies, mice were end pointed when tumor volumes exceeded 1500mm^3^ or when they showed significant respiratory distress from lung metastases.

### Human and murine ex vivo tumor models

To initiate the study, BALB/c mice were implanted with 3 x 10^5^ CT26.wt colon carcinoma cells subcutaneously. Once tumor volumes reached 1500 mm^3^, the mice were euthanized, and relevant tissues were extracted. For human tissue samples, tumor samples were collected from patients who had given informed consent and followed the Declaration of Helsinki guidelines during surgical resection. Collection of human tissue/fluid for this study was made possible by the Global Tissue Consent and Collection Program at the Ottawa Hospital Research Institute. The tissue collection program was approved by OHSN-REB under the protocol OHSN REB 20180079-01H. All tissues were sliced into 2mm sections and circular cores of 2mm diameter were extracted using a punch biopsy tool. These cores were then kept in a humidified incubator at 37°C and 5% CO2 in DMEM supplemented with 10% serum, 30mM HEPES, 1% (v/v) penicillin-streptomycin and 0.25 mg/L amphotericin B. The cores were treated with DMF for four hours and then infected with HSV.n212 at 3 x 10^4^ pfu/core. After 72 hours post-infection, fluorescence images were captured using the EVOS Live Cell Imaging System (Thermo Fisher) and cores were homogenized with a TissueLyser II (Qiagen) prior to titering.

### Immune profiling

#### Tissue processing

The tumors were dissociated utilizing the Miltenyi mouse tumor dissociation kit (Miltenyi Biotec, CA, USA, Cat. # 130-096-730) in conjunction with the gentleMACS Octo Dissociator (Miltenyi Biotec, Cat. # 130-096427). The spleens and tumor-draining lymph node (TdLN) (ipsilateral axillary, inguinal, cervical) were obtained and subjected to dissociation by mechanically crushing the organs through a 70 µm strainer, utilizing the plunger of a 3 mL syringe. The erythrocyte lysis of all dissociated spleens was performed using ACK buffer (Gibco, Cat. # A1049201). The cell suspensions were counted, and a total of 2 x 10^6^ cells were reconstituted in 200 µl of FACS buffer (0.5% BSA-PBS) before being transferred to round-bottom 96-well plates for the purpose of staining.

#### Flow cytometry

Following the aforementioned tissue processing protocol, the cells were exposed to staining using the fixable viability dye FVS510 (BD Biosciences, NJ, USA, Cat. #564406) at a dilution of 1:1000 in phosphate-buffered saline (PBS) for a duration of 30 minutes at the room temperature. The cells underwent a washing process and were subsequently subjected to incubation with anti-CD16/32 (1:100, BD Biosciences, Cat. # 553141) in a solution of 0.5% BSA-PBS for a duration of 30 minutes at a temperature of 40°C. This step was performed in order to prevent non-specific antibody binding to Fc receptors. The cells were subsequently subjected to staining using a specific subset of antibodies against the following targets: CD45-BV786 (1:1000, BD Biosciences, Cat. # 564225), CD3-AlexaFluor 700 (1:100, BD Biosciences, Cat. # 561805), CD4-V450 (1:1000, BD Biosciences, Cat. # 560468), CD8aPE-CF594 (1:100, BD Biosciences, Cat. # 562283), CD25-PE (1:100, Thermo Scientific, Cat. # 12-0251-82), CD69-BV605 (1:100, BD Biosciences, Cat. #563290), CD44-APC (1:100, BD Biosciences, Cat. #563058), CD62L-FITC (1:100, BD Biosciences, Cat. #553150), PD1-APC (1:100, BD Biosciences, Cat. # 562671), CD127-PE-Cy7 (1:100, BD Biosciences, Cat. # 560733), CD49b-BUV395 (1:100, BD Biosciences, Cat. # 553857), CD11b-APC-Cy7 (1:200, BD Biosciences, Cat. #553312), CD11c-PE (1:100, BD Biosciences, 553802), CD86-APC-R700 (1:100, BD Biosciences, Cat. # 565479), IA/IE-BV605 (1:200, BD Biosciences, Cat. # 563413), CD19 (1:100, BD Biosciences, Cat. # 553785), F4/80-BUV605 (1:100, BioLegend, CA, USA, Cat. #123118). The PD-L1-APC-Cy7 antibody (BioLegend, Cat. # 124313) was used at a dilution of 1:100. The cells were subsequently rinsed and reconstituted in a solution containing 1% paraformaldehyde (PFA) in phosphate-buffered saline (PBS). The acquisition of samples was conducted using the BD LSRFortessa™ instrument in the Flow Cytometry and Cell Sorting core facility of the Ottawa Hospital Research Institute. The data underwent analysis using FlowJo v10.8 software. Unstained controls and fluorescence-minus-one (FMO) controls were made concurrently. Compensation was performed using Ultracomp eBeads (Thermo Scientific, Cat. # 01-2222-42), which are single-stained beads were used for compensation.

### Statistics

GraphPad Prism were used in all graphs and statistical tests. Individual statistical tests were detailed in figure legends. Two-tailed unpaired Student’s t-test was used when means of two groups were compared. One-way ANOVA with Dunnett’s or Tukey’s multiple correction test were used when means of more than two groups were compared. Analysis of *in vivo* survival data was performed by the Kaplan-Meier method followed by log-rank test. Biological replicates are indicated by a number n, and error calculated as the standard error of the mean (SEM) For all analyses, *p < 0.05, **p < 0.01, ***p < 0.001, ****p < 0.0001; n.s. = not significant. Data were reproduced by at least two different operators.

## Results

### Dimethyl fumarate sensitizes cancer cells to HSV-1 infection

**Figure 1:**
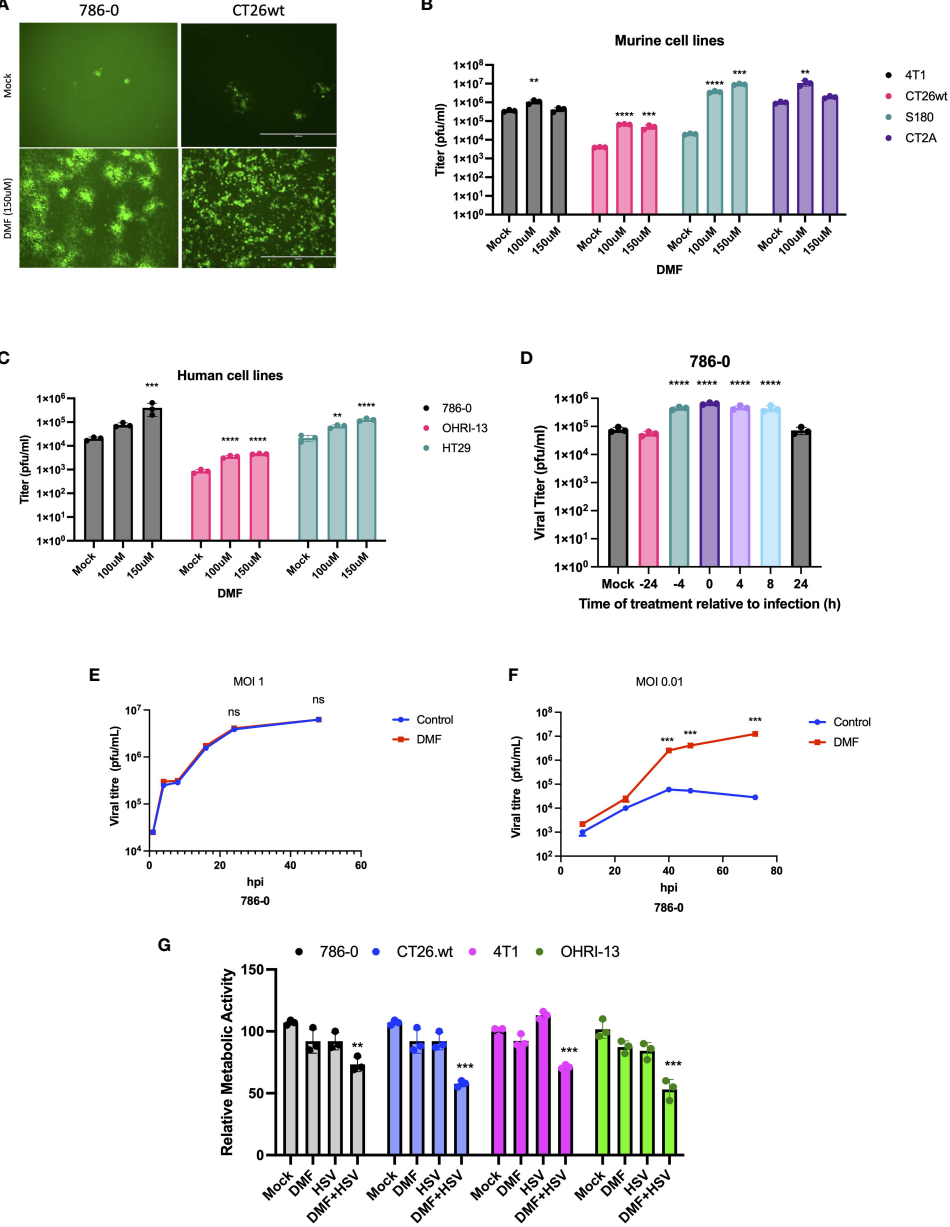
DMF sensitizes human and murine tumor types to HSV-1. **(A-C)** Various human and murine cell lines were pre-treated with DMF (100, and 150uM), then infected with HSV.n212 (MOI 0.01). Forty-eight hours after infection, fluorescence images were taken from the infected cancer cells, as shown in **(A)** infectious viral particles were quantified by plaque assay as shown in B&C (N=3 mean ± SEM; one-way ANOVA with Dunnett’s multiple comparisons test compared to Mock for each cell line). **(D)** 786-0 cells were treated with 150 μM of DMF at various times before or after infection with HSV.n212 (MOI: 0.01) or untreated. Samples were collected 48 hours after infection and tittered by plaque assay (N=3 mean ± SEM; one-way ANOVA with Dunnett’s multiple comparisons test compared to Mock for each cell line). **(E, F)** Multistep (MOI, 0.01) and single-step (MOI, 3) growth curves. 786-0 cells were pretreated with DMF and infected with HSV.n212 at an MOI of 0.01, or 3; samples were tittered by plaque assay (n = 3; mean ± SD; two-tailed t-test). **(G)** 786-0 and other cancer cell lines indicated were pre-treated with DMF with different concentrations for 4 hours and then infected with HSV.n212 at an MOI of (0.01). Seventy-two to ninety-six hours post-infection cytotoxicity was assessed by incubating samples with Resazurin metabolic dye for 150 minutes at 37C before measuring fluorescence (530nm excitation, 590 nm emission). Values were normalized to that of untreated control (N=3 mean ± SEM; **p < 0.01, ***P < 0.001, and ****p < 0.0001; one-way ANOVA with Dunnett’s multiple comparisons test compared to Mock for each cell line).

The main objective of our study was to evaluate the potential applicability of DMF to HSV-1-based oncolytic virus platforms. Consequently, we first investigated the effects of DMF on HSV-1 replication in various murine and human cancer cell lines. As a starting point, human renal 786-0 carcinoma cells, which are typically resistant to oncolytic virus infection, underwent a pre-treatment phase of 4 hours with DMF as per previous studies (24). Subsequently, these cells were infected with HSV.n212 that was genetically modified to express green fluorescent protein, at a low multiplicity of infection (MOI), as shown in (**Figure 1A**). An increase in fluorescence upon pretreatment with DMF and infection with HSV.n212 was observed in 786-0 cells as well as in murine CT26.wt colon carcinoma cells which are HSV-resistant at baseline and syngeneic in Balb/c mice (**Figure 1A**). In addition, fluorescent microscopy confirmed increased HSV.n212-GFP transgene expression in murine CT26.wt, CT2A glioma,S180 sarcoma and human 786-0 cells as shown in (**Supplementary Figures S1A, B**). Subsequently, the viral sensitizing potential of DMF in impacting HSV.n212 viral titers was evaluated in several cancer subtypes including breast (murine 4T1), colon (murine CT26.wt, human HT29), glioma (murine CT2A), sarcoma (murine S180), renal (human 786-0) and melanoma (patient derived OHRI-13) where we observed a significant increase compared to HSV.n212 alone (**Figures 1B, C**). This viral enhancing effect was also observed when DMF was administered concurrently with the virus as well as with post-treatment times as long as 8 hours as shown in (**Figure 1D**). In addition, we conducted assessments with various strains of HSV-1 to ascertain the generalizability of DMF’s impact across diverse oncolytic mutants. Our findings revealed a consistent increase in titer of other strains of HSV including HSV.G47Δ, HSV.d810 and HSV.γ34.5 within distinct cancer models, as shown in (**Supplementary Figures S2, 3**), affirming the broad applicability of DMF’s efficacy across oHSV-1 strains. Furthermore, we looked into the ability of DMF to potentiate HSV-1 infectivity by comparing multi-step to single-step growth curves. Similar to what we observed with VSV, DMF was able to strongly improve HSV.n212 when infected at a low MOI of 0.01, but not at a high MOI of 3 through quantification of titer by plaque assay as illustrated in (**Figures 1E, F**). This suggests that DMF promotes viral spread to increase its growth, but not through increasing the rate of HSV-1 replication or viral entry. To further assess the oncolytic impacts of HSV.n212 in the presence of DMF, we pretreated cancer cells with DMF before infection with HSV.n212, and cell viability was assessed 72 hours after infection. Combined treatment with DMF and HSV.n212 resulted in a significant (25-50%) decrease in cell viability in human 786-0 as well as several other human and murine cell lines (**Figure 1G**). To confirm the synergetic effect of the combination therapy, an analysis of non-constant combinations was conducted, focusing on the interaction between HSV.n212 and DMF compared to their respective monotherapies. This analysis is detailed in (**Supplementary Table S1**), which presents the Combination Index Score (CSI) consistently between 0.7-0.8 in CT26.wt cells, confirming a synergistic interaction.

### Dimethyl fumarate enhances HSV-1 viral infection in a various of ex vivo tumor models

**Figure 2:**
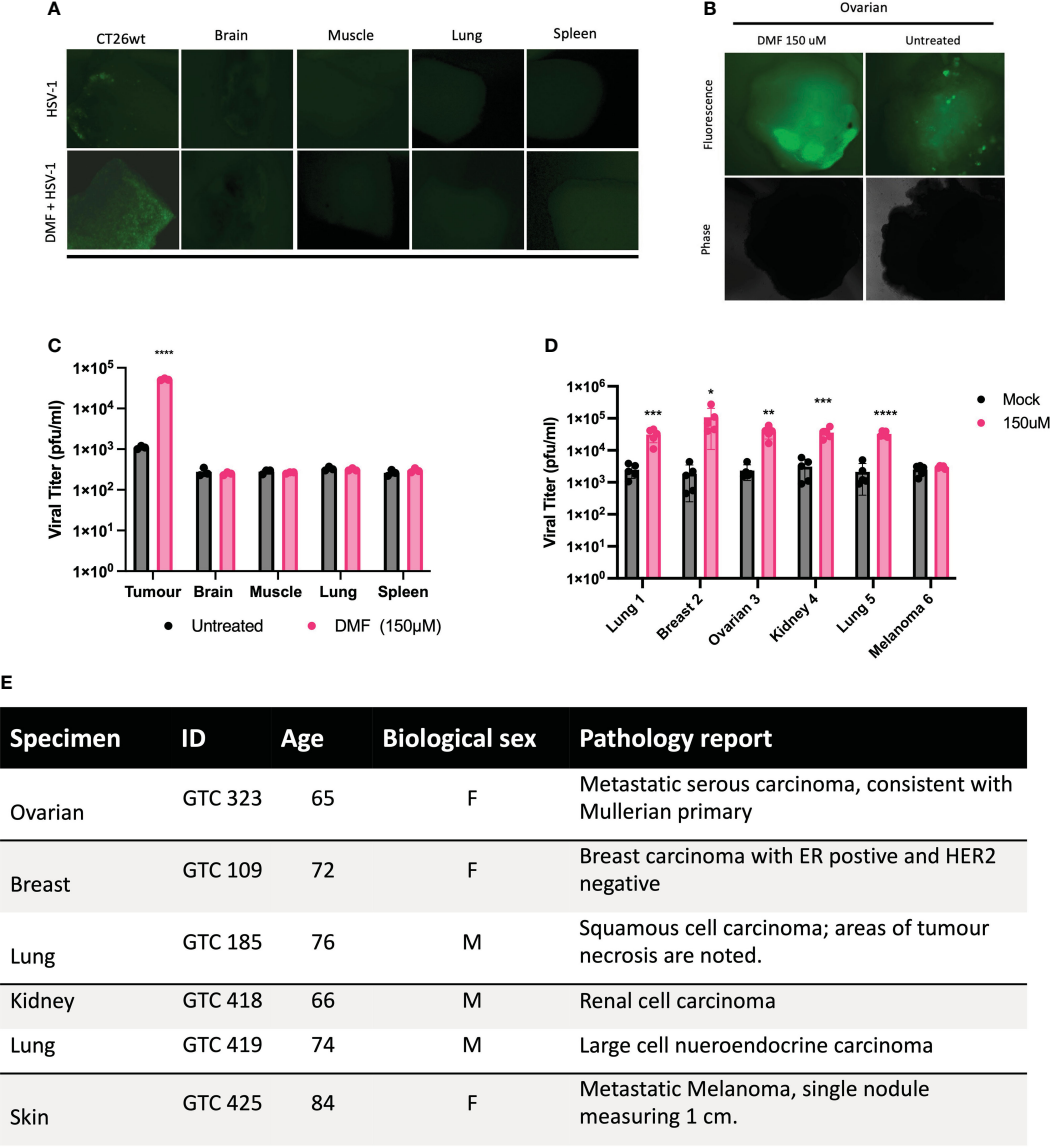
DMF enhances the replication of HSV.n212 in ex vivo tumor tissues and patient-derived explants. **(A, B)** CT26.wt (murine colon carcinoma) tumors were grown in BALB/c mice until they reached a volume of 1500mm^3^. Tissues were collected, cored, and treated for four hours with DMF at indicated concentrations prior to infection with 3x10^10^ plaque-forming units of HSV.n212. To monitor viral infection, GFP images were captured, and supernatants were collected at 72 hpi. Representative GFP images are shown for tumor and normal tissues as in **(B)** Infectious viral particles were quantified by standard plaque assay **(B)**. **(C, D)** Clinical human samples were obtained and cored. Cores were treated with DMF (150μM) for 4 hours, then infected with HSV.n212 (3 x 10^4^ pfu/core). At 72hpi, representative fluorescent images were obtained. Infectious viral particles were quantified by standard plaque assay (n>5; ns = no significance, *P< ****P<0.0001 by two-tailed t-test). **(E)** A table presents clinical characteristics of patient-derived tumor specimens used in this study. (n>5, mean ± SD; ns = no significance, *P< 0.05, **p < 0.01, ***p < 0.001 and ****P<0.0001 by two-tailed t-test).

The inherent selectivity of oncolytic virotherapy towards cancer tissues is one of its advantages over conventional therapies. Therefore, the effect of DMF on oncolytic HSV-1 selective infectivity was evaluated to confirm that DMF does not enhance the replication of the virus in normal tissues. Tumor cores from mice subcutaneously implanted with CT26.wt murine colon cancer cells, as well as cores from normal tissues such as brain, spleen, muscle, and lung, were collected and subsequently infected with HSV.n212 in the presence or absence of DMF (150 µM). (**Figure 2A**) shows representative fluorescence images of cores that were pretreated with DMF prior to HSV.n212 infection. Images and corresponding viral titers (**Figure 2B**) show a clear enhancement of HSV.n212 following pretreatment with DMF in CT26.wt cores. In sharp contrast, no enhancement of HSV.n212 was observed in normal brain tissues and other normal tissues, indicating that the selectivity of the virus towards the tumor is still maintained regardless of DMF pretreatment. Furthermore, similar results were observed in cores obtained for S180 and CT2A tumors, as shown in (**Supplementary Figure S4**). When we tested our combination strategy in primary human *ex vivo* clinical samples, DMF increased HSV.n212 infection across a large variety of tumor types, including breast, lung, and ovarian, as demonstrated by viral plaque assay and fluorescence microscopy (**Figures 2C-E**). In an ovarian cancer sample, DMF increased the viral titer by over 14-fold. Of 6 human cancer specimens tested, only a melanoma sample was found to be non-responsive to DMF even though baseline infection/viral titer was similar across all samples. Together, these results demonstrate that DMF’s ability to increase HSV.n212 viral infectivity is selective and applicable across different types of human and murine tumors.

### FMAEs promote HSV-1 infection

**Figure 3:**
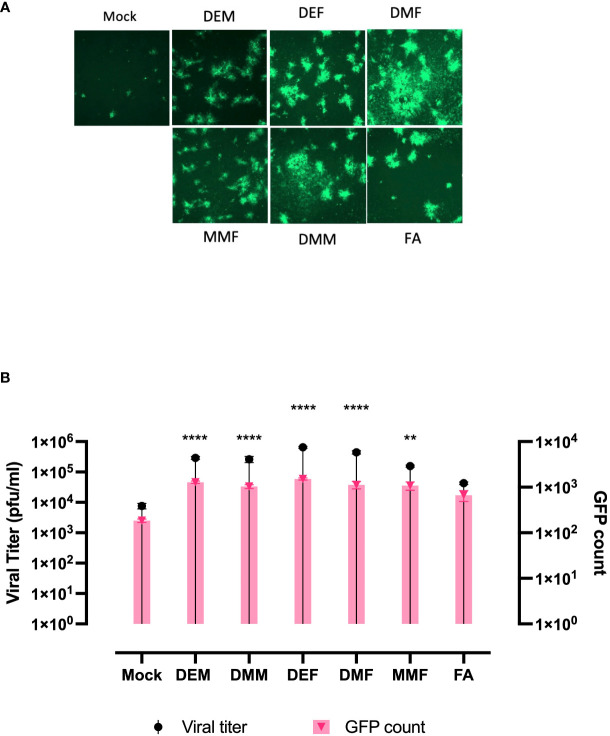
FAMEs promote infection by HSV.n212 in 786-0 cells were pretreated with various FMAEs and analogs for 4 hours and subsequently infected with oncolytic HSV.n212 expressing GFP at **(A)** an MOI of 0.01. Seventy-two hours after infection, we obtained fluorescence images of the infected 786-0 cells. **(B)** Corresponding viral titers were determined from supernatants 72 hours after infection. (N=3 mean ± SEM; one-way ANOVA with Dunnett’s multiple comparisons test compared to Mock for each cell line). **p < 0.01 and ****p < 0.0001.

Apart from DMF, a number of fumaric and maleic acid esters (FMAEs) exhibit properties that reduce inflammation and regulate the immune response. In light of this, we investigated whether other FMAEs and their cis- and trans-isomers (maleic acid esters) had an impact HSV.n212 infection of cancer cells, comparable to what was previously shown with VSV (24). Indeed, dimethyl maleate (DMM), diethyl maleate (DEM), and diethyl fumarate (DEF) pretreatment led to strong HSV.n212 infection and oncolysis within 786-0 cells *in vitro* (**Figures 3A, B**). Additionally, MMF, the biologically active metabolite of DMF, did enhance HSV.n212 as shown in (**Figures 3A, B**). While some enhancement in HSV.n212 spread and titer (**Figures 3A, B**) was observed when pre-treating 786-0 with fumaric acid (FA), a compound that does not easily traverse the cell membrane, this did not reach statistical significance. Collectively, our findings suggest that the use of DMF and other FMAEs, characterized by their high bioavailability, can significantly enhance the growth of HSV-1 in 786-0 cells.

### DMF suppresses the antiviral response

**Figure 4:**
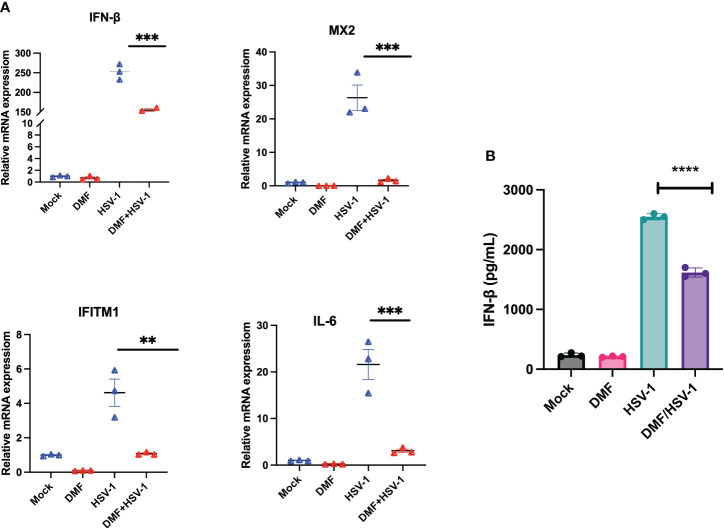
DMF increases HSV.n212 infection via IFN-1 inhibition. **(A)** 786-0 were pretreated with Mock or DMF (150uM) for 4h, after which time cells were washed and infected with HSV-1 MOI 0.1 or mock infected. 24 hour later, RNA was collected, converted to cDNA and qRT-PCR was performed. Data represented the normalized fold change. **(B)** 786-0 cells were treated as in **(A)** and at 36 hours post-infection, supernatants were collected and assayed by ELISA for IFN-β levels (N=3 mean ± SEM; *P < 0.05, **p < 0.01, ***P < 0.001 and ****p < 0.0001. one-way ANOVA with Dunnett’s multiple comparisons test compared to Mock for each cell line).

To further assess if DMF treatment suppresses innate immunity induced by oncolytic HSV-1 as previously observed in the context of VSV (24), IFN-β and downstream interferon-stimulating genes (ISGs) were investigated at the mRNA level. 786-0 cells were pretreated with either mock or DMF (150 µM) and four hours later infected with HSV.n212, or mock infected. 24 hour later, RNA was collected, converted to cDNA and qRT-PCR was performed. All genes, including IFN-β, MX2, IFITM1, and IL6, were significantly suppressed with the combination treatment in comparison with HSV.n212 alone as shown in (**Figure 4A**). This suggests that DMF increases HSV.n212 infection via IFN-1 inhibition analogous to what was previously observed with oncolytic VSV. Quantification of IFN-β secretion 24 hpi by enzyme-linked immunosorbent assay (ELISA) followed a similar trend as shown in (**Figure 4B**).

### DMF enhances the therapeutic effectiveness of oncolytic HSV.n212

Given that DMF is a clinically approved drug, and it broadly and robustly enhances the growth and activity of oncolytic HSV-1 in several human and murine cancer cells with conversely limited effects on normal tissues, we next evaluated the potential therapeutic benefit of combining DMF with oncolytic HSV-1 *in vivo*. The primary aim of the first experiment was to determine the optimal dosage of HSV.n212 that would have a significant effect on the progression of tumors. We administered HSV.n212 intratumorally up to six times in the CT26.wt model. We saw that infection with HSV.n212 administered either three or six times resulted in regression of the tumors as compared to the monotherapy at lower frequencies or with PBS treatment. A survival study of this experiment shows that CT26.wt-bearing mice having received HSV.n212 either 3x or 6x had significantly prolonged survival compared to PBS (HSV-1 3x: P = 0.019 vs. PBS group; HSV-1 6x: P = 0.013 vs. PBS group) (**Supplementary Figure S5**).

We next sought to determine the most effective route of administration for DMF. DMF has received approval from the U.S. Food and Drug Administration (FDA) for oral administration to patients with multiple sclerosis, which is convenient, but which may not sufficiently reach the tumor. Conversely, administration through intratumoral injection can be more effective by directly impacting the tumor microenvironment, offering opportunities for co-administration as HSV-1 is currently approved for applications where the virus is administered intra-tumourally.

The CT26.wt model was used to assess the efficacy of oral and intratumoral (i.t) routes of delivery of DMF in achieving tumor control in combination with HSV-1. In our study, we had four distinct groups, including DMSO, HSV.n212 alone, DMF (i.t.)/HSV.n212, and DMF (gavage)/HSV.n212. CT26.wt tumor-bearing mice received DMF either intratumorally or by oral gavage (200mg/kg). Five hours later, a bolus of 25μL PBS containing 1 x 10^8^ pfu of HSV.n212 was injected intratumorally. This treatment was repeated two more times, with a one-day interval between each treatment. While both routes of administration led to delayed tumor progression in combination with HSV.n212, delivering DMF and HSV i.t. had the most robust effect, leading to some long-lasting remissions (approximately 10%) as shown in (**Supplementary Figure S6**).

**Figure 5:**
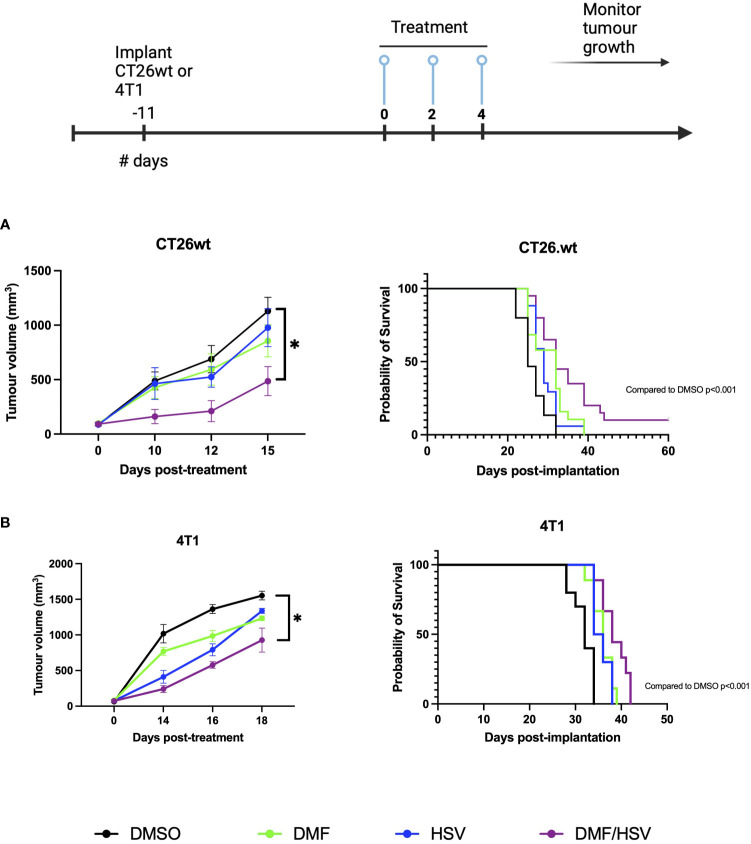
DMF improves HSV.n212 therapeutic efficacy in murine *in vivo* tumor models. BALB/c nude mice were implanted with 3 × 10^5^ CT26.wt cells or 5 × 10^5^ 4T1 cells. Upon reaching ~ 100mm^3^, mice were treated three times every other day with the regimen of 200 mg/kg DMF or DMSO (intratumorally) followed by HSV.n212 (1E8 pfu) or PBS (intratumorally) 5 hours later. Tumor volumes were monitored every 2-3 days (n > 15, mean ± SEM; *P<0.05, **P<0.01 by one-way ANOVA). Mice were culled when tumor volumes reached 1500mm^3^ for survival analysis. Kaplan-Meier curves were plotted and compared using the log-rank (Mantel-Cox) test (CT26.wt: DMSO = 15, DMF = 19 HSV.n212 = 18, combo = 20; 4T1: DMSO = 10 DMF = 9, HSV.n212 = 10, combo = 9).

Having established that intratumoral administration of DMF over gavage results in better survival rates in combination with i.t. HSV.n212, our subsequent objective was to more robustly investigate the efficacy of DMF (intratumoral) in combination with HSV.n212 compared to other monotherapies, including HSV.n212, DMF, and DMSO. Treatments were tested in two different murine models of cancer: CT26.wt colon and 4T1 breast carcinoma. Mice given the combination therapy after tumors reached ~100mm^3^ in size three times every other day (days 0, 2 and 4) showed a significant regression in tumor progression when compared to monotherapies in both the CT26.wt and 4T1 model. When looking at survival data in these models, the combination therapy significantly prolonged survival compared with either monotherapy (combination therapy compared to DMSO alone P = 0.001; HSV.n212 alone: P = 0.006, DMF alone: p = 0.03) (**Figure 5A**). In the more aggressive 4T1 model, the DMF/HSV.n212 combination significantly increased survival in comparison to all other conditions (combination therapy compared to DMSO alone P = 0.001; HSV.n212 alone: P = 0.01, DMF alone: p = 0.02) (**Figure 5B**). These results demonstrate that the combination of DMF and HSV.n212 therapy results in a greater therapeutic benefit compared to placebo or monotherapy in two different mouse models of cancer.

### Effects of HSV-1/DMF combination therapy on tumor immune profile

**Figure 6:**
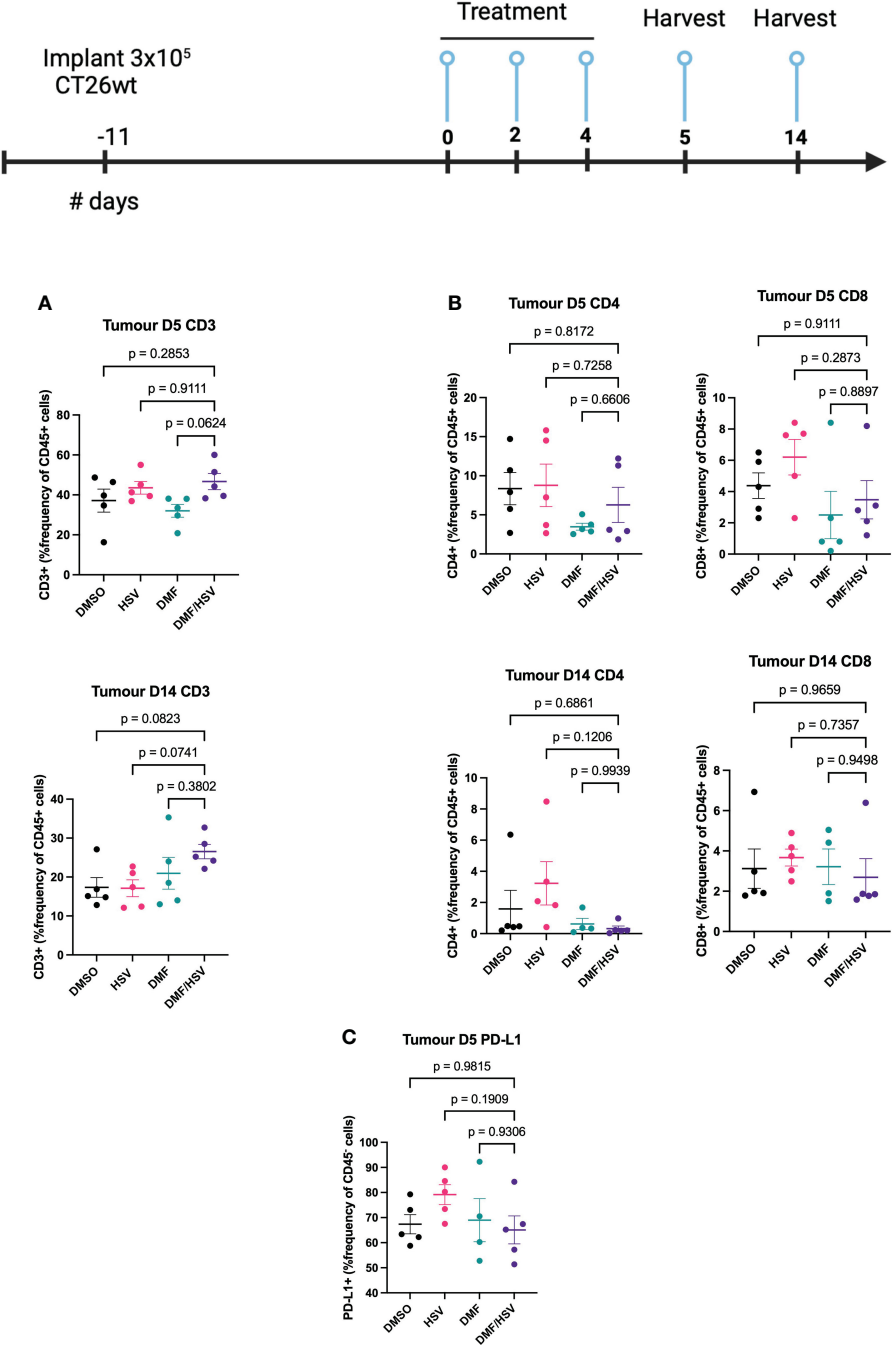
FACS analysis of combination treatment in CT26.wt model. BALB/c mice were implanted with 3 × 10^5^ CT26.wt cells. Upon reaching ~ 100mm^3^ mice were treated three times every other day with the regimen of 200 mg/kg DMF or DMSO 4 h later (intratumorally) followed by HSV.n212 (1E8 pfu) or PBS (intratumorally). On day 5 and 14 post-treatment, mice were sacrificed, and tumors, spleen and lymph nodes harvested, cells were dissociated and stained with fluorochrome-conjugated anti-mouse antibodies, and multicolor FACS was performed. **(A)** Tumor-infiltration in total CD45^+^CD3^+^ T-cell populations **(B)** Tumor-infiltration total CD45^+^ CD4+ and CD8+ T-cell populations on day 5 and 14 post treatment. **(C)** PD-L1^+^ in CD45^-^ population within tumor microenvironment. (N=5 mean ± SEM; one-way ANOVA with Dunnett’s multiple comparisons test compared to Mock for each cell line).

In order to provide a more comprehensive understanding of the immune response subsequent to therapy in the CT26.wt cancer model, mice were subjected to euthanasia on days 5 and 14 subsequent to the first administration of the treatment dosage (as per i.t dosing regimen described above). Tumors, tumor-draining lymph nodes (TdLN), and spleens were collected for the purpose of assessing cell-mediated immune responses. We detected an increase in the CD3+ population inside the tumor compartment of mice treated with the combination regimen compared to all other groups by day 14; however, this did not reach statistical significance comparing to individual groups in *post-hoc* tests (ANOVA p=0.09, **Figure 6A**). Notably, there was no concurrent increase detected in the CD4 and CD8 at any time point, suggesting this could be attributable to a rise in double-negative T-cells (**Figure 6B**). Notably, we also observed elevated expression levels of PD-L1 on the CD45^-^ population which are predominantly CT26.wt cells as shown in (**Figure 6C**).

## Discussion

The obstacle presented by the resistance of tumors to oncolytic virotherapy is a well-recognized problem that hampers the achievement of clinical efficacy. Several research groups including ours have dedicated efforts towards developing strategies involving small molecules to enhance the susceptibility of tumors to oncolytic virus infection (12). DMF arises as a versatile therapeutic candidate in the field of oncology due to its multifaceted characteristics, which include potent anti-tumor properties, immunomodulatory capabilities, and FDA-approved status (26). A recent study conducted by our team has demonstrated that DMF can improve the effectiveness of VSVΔ51 in a variety of preclinical models (24). Despite the commercial failure of Imlygic, HSV-1 remains a leading candidate in the OV field and holds much promise in the field of cancer therapy due to its ease of genetic manipulation, its ability to specifically target and eliminate cancer cells, and induce immune responses against tumors. Additionally, HSV-1 continues to stand as the only platform that has successfully transitioned into clinical use in North America, Europe and most recently Japan (4). In this study, we examined the effects of DMF and various FMAEs on oncolytic HSV-1 in the context of cancer.

In the present investigation, our findings demonstrate that the utilization of the clinically approved DMF leads to a notable augmentation of HSV-1 propagation, evident through increased expression of virally encoded green fluorescent protein (GFP) across a variety of HSV-1 mutant strains (**Figures 1A**, **Supplementary Figures S2, 3**). This results in enhanced viral output, as well as significantly enhanced oncolytic effects within multiple human and murine cancer cell lines (**Figures 1B, C, G**). In addition to DMF, several FMAEs demonstrated a consistent effect on HSV.n212, resulting in an increase in both GFP expression and viral titers in 786-0 cells (**Figure 3**). Importantly, these outcomes align closely with our prior observations involving VSVΔ51 (24).

DMF exerts inhibitory effects on the cellular response to type I IFN by, in part, reducing IFN production through the modulation of NFκB (27). Additionally, DMF hinders the activation of signal transducer and activator of transcription 1 (STAT1) following IFN stimulation. Indeed, as we observed in the context of oncolytic VSV, a significant decrease in the expression of interferon stimulating genes was observed following DMF treatment and HSV.n212 infection (**Figure 4**).

In this study, we also optimized an intratumoral HSV.n212 dosage regimen in a subcutaneous CT26.wt murine tumor model. We found that administration of three- and six-times HSV.n212 every other day similarly reduced tumor growth compared to lower frequency regimens (**Supplementary Figure S5**). Consequently, we proceeded with the three-dose infection regimen.

Given the current practice of oral DMF delivery in MS patients, we investigated whether gavage DMF could match intratumoral injection, which was used in our previous study with VSV. While DMF administered by gavage led to a delay in tumor growth, intratumoral DMF delivery surpassed gavage in tumor management in combination with HSV.n212 (**Supplementary Figure S6**). Several preclinical studies have used oral DMF as a monotherapy for cancer with favorable findings (28, 29). However, these studies used frequent and lengthy dosing strategies, at lower concentrations, which may explain the difference in results.

We chose the CT26.wt model as a starting point to evaluate the combination of HSV-1 and DMF in this study owing to its relative resistance to OVs *in vivo*, and *in vitro* and ex vivo responsiveness to DMF with respect to enhancing OV spread. As predicted, the combination of DMF and HSV.n212 resulted in a significant delay in tumor growth, surpassing the effect of either monotherapy (**Figure 5**). While neither monotherapy led to long-term tumor control, the combination strategy demonstrated a 10% cure rate within the CT26.wt model, aligning with prior findings reported with oncolytic VSV (cure rate of 20%) (24). We also evaluated the combination therapy in a more aggressive 4T1 model. In this case, the combination therapy significantly extended survival and reduced tumor growth but did not lead to long term cures.

The compelling findings reported in our study of the combination of DMF with oncolytic HSV-1 in two different tumor murine models underscore the potential of this strategy as a therapeutic avenue.

While further dose optimization experiments will be required, the current study suggests that a combinatory approach involving the administration of DMF intratumorally ahead of HSV.n212 (5h apart), administered a total of three times every other day can be effective for tumor control. Nevertheless, it is apparent that additional efforts are required to enhance the effectiveness of this therapy. Thus, we examined the treatment’s immunological effects in CT26.wt to determine if they were linked to tumor control. Remarkably, a significant increase in CD3 cell counts was found in response to the combined therapy (**Figure 6A**); however, there was no concurrent rise in the populations of CD4 and CD8 cells. This suggests an increase in double-negative T-cells, however further studies will be needed to determine whether these are beneficial or not to tumor control, as these have been reported to either play a pro or anti-tumor role depending on the context and tumor microenvironment (30). The anti-inflammatory effects of DMF are well-known which might overall dampen lymphocyte responses despite greater infiltration of CD3+ cells (31). While outside the scope of the current study, this suggests that a potential avenue for exploration entails the use of oncolytic HSV-1 candidates that have been genetically modified to express therapeutic genes capable of stimulating immunological responses. Based on our current findings, strategies that could capitalize on the infiltration of double-negative T-cells or conversely enhance CD4+/CD8+ recruitment to the tumor may be beneficial, as may be strategies to dampen the PD-1/PD-L1 axis.

Moreover, it is noteworthy that the CT26.wt model exhibits a notable expression of PD-L1 in CD45^-^ populations (tumor cells) (**Figure 6C**). In this particular situation, it is plausible that the effectiveness of the combination approach could be enhanced by integrating an immune checkpoint inhibitor that specifically targets PD-L1, hence amplifying immune responses.

In short, this work shows that the clinically approved DMF increases HSV-1 propagation in several human and murine cancer cell lines. In the immune competent murine CT26.wt colon cancer and 4T1 breast cancer models, we showed that the DMF-HSV-1 combination therapy significantly reduced tumor growth more than respective monotherapies. Altogether, our findings suggest that combination approaches using DMF and engineered HSV-1 that can maximize anti-tumor immune response warrant further testing.

## Data availability statement

The raw data supporting the conclusions of this article will be made available by the authors, without undue reservation.

## Ethics statement

The studies involving humans were approved by The Global Tissue Consenting committee, Ottawa Hospital Research Institute. The studies were conducted in accordance with the local legislation and institutional requirements. The participants provided their written informed consent to participate in this study. The animal study was approved by Animal Care Committee, University of Ottawa. The study was conducted in accordance with the local legislation and institutional requirements.

## Author contributions

AA: Conceptualization, Data curation, Formal Analysis, Investigation, Writing – original draft, Writing – review & editing. ZT: Data curation, Formal Analysis, Investigation, Writing – review & editing. MT: Investigation, Writing – review & editing. AC: Data curation, Writing – review & editing. BW: Validation, Investigation, Writing – review & editing. RA: Conceptualization, Supervision, Writing – review & editing, Project administration. J-SD: Conceptualization, Funding acquisition, Project administration, Supervision, Writing – review & editing.
