# Supplementary material for: Unlocking the potential of dimethyl fumarate: enhancing oncolytic HSV-1 efficacy for wider cancer applications
Source: Front Immunol. 2023 Dec 19;14:1332929. doi: 10.3389/fimmu.2023.1332929 (PMC10758402; doi:10.3389/fimmu.2023.1332929)
Supplement: Supplementary file 1 [file DataSheet_1.pdf]

Supplementary Material

Unlocking the Potential of Dimethyl Fumarate: Enhancing Oncolytic HSV-1 Efficacy for Wider Cancer Applications

Akram Alwithenani[1,2,3], Zaid Taha[1,2], Max Thomson[1], Andrew Chen[1], Boaz Wong[1,2], Rozanne Arulanandam[1], Jean-Simon Diallo[1,2]

[1]*Centre for Cancer Therapeutics, Ottawa Hospital Research Institute, Ottawa, Ontario, K1H 8L6, Canada.*

[2]*Department of Biochemistry, Microbiology, and Immunology, Faculty of Medicine, University of Ottawa, Ontario, K1H 8M5, Canada*

[3]*Department of Clinical Laboratory Science, Faculty of Applied Medical Science, Umm Al-Qura University, Makkah, Saudi Arabia*

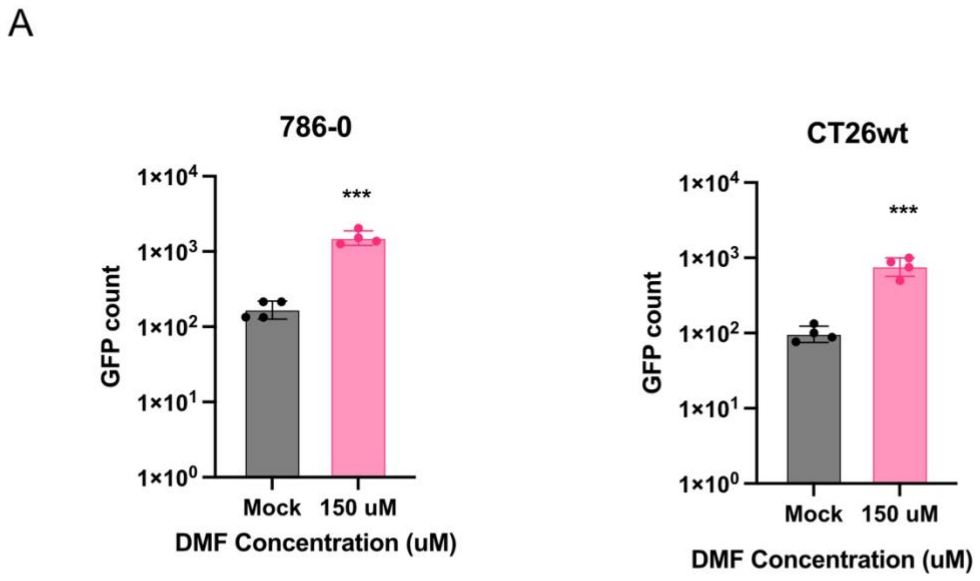
A
786-0
CT26wt
GFP count
***
1×10^4
1×10^3
1×10^2
1×10^1
1×10^0
Mock
150 uM
DMF Concentration (uM)

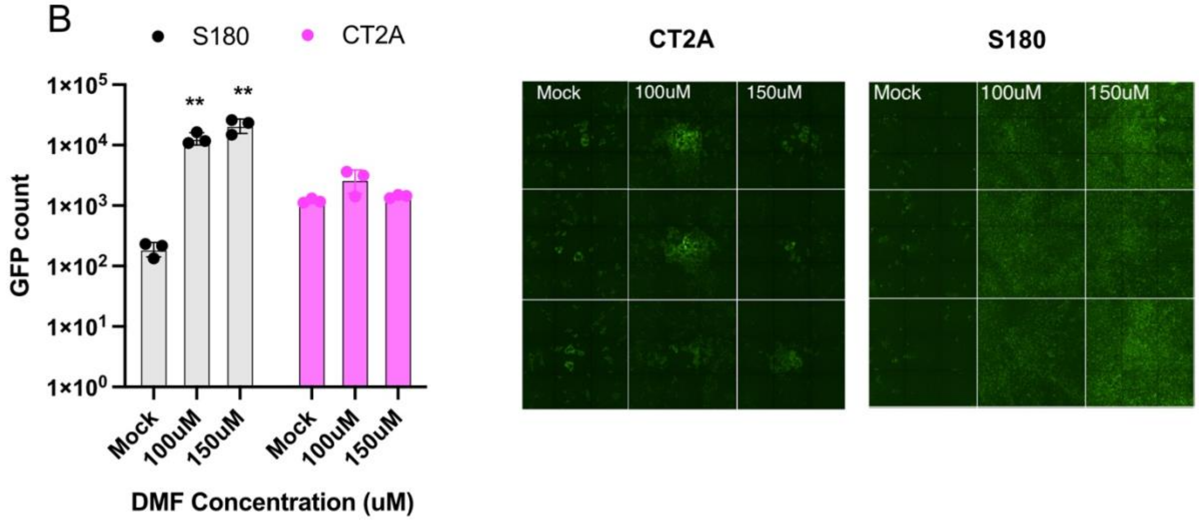
B
S180
CT2A
GFP count
**
1×10^5
1×10^4
1×10^3
1×10^2
1×10^1
1×10^0
Mock
100uM
150uM
DMF Concentration (uM)
CT2A
S180

Figure S1: DMF enhances GFP of HSV.n212 in Several cancer cell lines. (A) representative GFP counts and images of 786-0 and CT26.wt cells upon treatment as described in Figure 1. (n = 3; mean ± SD; two-tailed t-test; *P < 0.05, ***P < 0.001). **(B)** GFP count and florescent images of CT2A and S180 Cells upon treatment as described in Figure 1. [n = 3; mean ± SD; *P < 0.05, ***P < 0.001, one-way analysis of variance (ANOVA), as compared to the untreated condition].

Table S1: Synergistic Effect of DMF + HSV.n212 in CT26.wt Model. This table presents the Combination Index Score (CSI) for the combined treatment of DMF and HSV.n212, specifically in their capacity to induce cell death. The data was analyzed utilizing the Compusyn software. The computation of the combination index was executed through a predictive algorithm. Notably, a score below 1 within this framework signifies a synergistic effect under the specified condition.

Dose DMF (uM)	Dose HSV.n212 (MOI)	Effect	CI
**50**	0.01	0.85	0.72
**100**	0.01	0.66	0.71
**150**	0.01	0.48	0.77
**222**	0.01	0.2	0.72

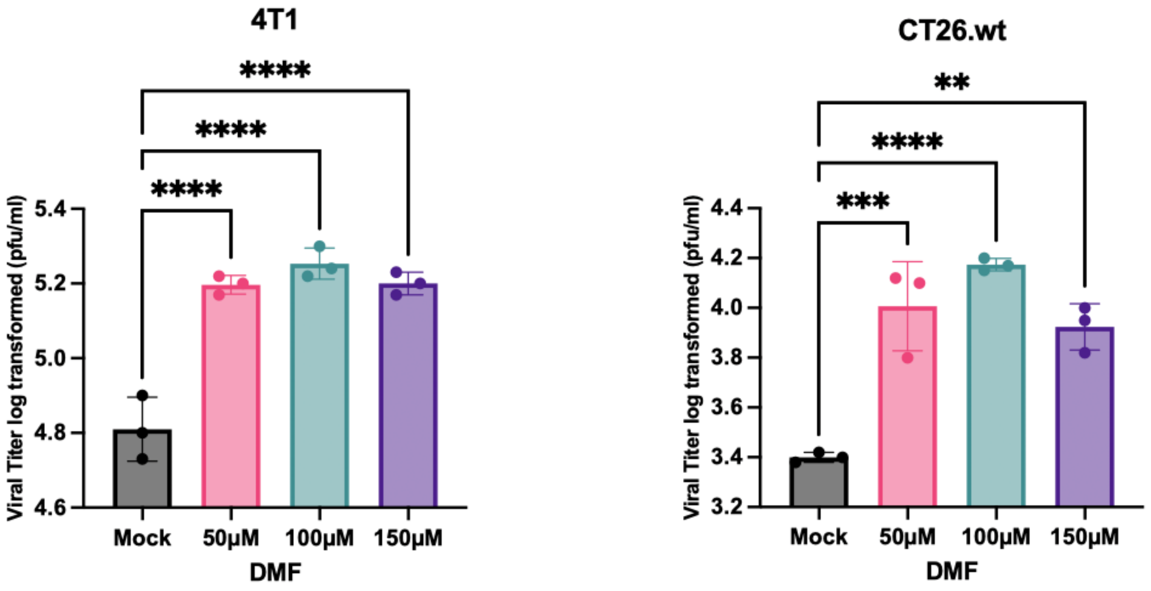

4T1
****
****
****
Viral Titer log transformed (pfu/ml)
5.4
5.2
5.0
4.8
4.6
Mock
50μM
100μM
150μM
DMF
CT26.wt
**
****
***
Viral Titer log transformed (pfu/ml)
4.4
4.2
4.0
3.8
3.6
3.4
3.2
Mock
50μM
100μM
150μM
DMF

Figure S2: DMF enhances HSVd810. Murine mammary carcinoma (4T1) and murine colon carcinoma (CT26.wt) cell lines were pretreated with DMF at various concentrations (0uM, 50uM, 100uM, 150uM) and four hour later infected with HSV.d810 at MOI of (0.1). Corresponding viral titers were determined 48 hours after infection from pellets. Florescence images were taken from all conditions. [n = 3; mean ± SD; *P < 0.05, ***P < 0.001, one-way analysis of variance (ANOVA), as compared to the untreated condition].

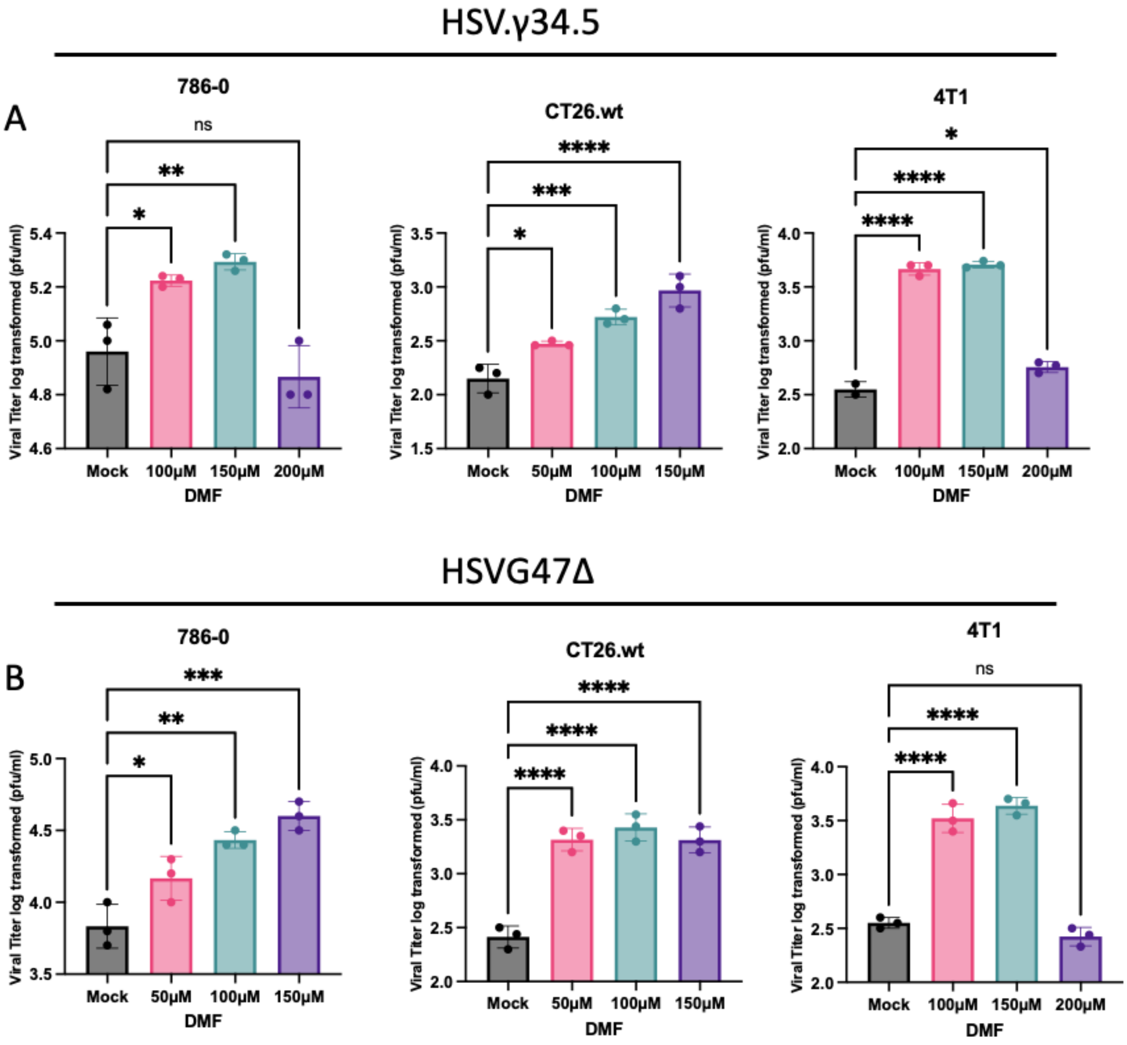
HSV.γ34.5
A
786-0
ns
**
*
Viral Titer log transformed (pfu/ml)
5.4
5.2
5.0
4.8
4.6
Mock
100μM
150μM
200μM
DMF
CT26.wt
****
***
*
3.5
3.0
2.5
2.0
1.5
Mock
50μM
100μM
150μM
DMF
4T1
*
****
****
4.0
3.5
3.0
2.5
2.0
Mock
100μM
150μM
200μM
DMF
HSVG47Δ
B
786-0
***
**
*
5.0
4.5
4.0
3.5
Mock
50μM
100μM
150μM
DMF
CT26.wt
****
****
****
4.0
3.5
3.0
2.5
2.0
Mock
50μM
100μM
150μM
DMF
4T1
ns
****
****
4.0
3.5
3.0
2.5
2.0
Mock
100μM
150μM
200μM
DMF

Figure S3: DMF enhances HSVγ34.5 and HSVG47Δ. Murine mammary carcinoma (4T1) and other murine and human cell lines were pretreated with DMF at various concentrations (0uM, 50uM, 100uM, 150uM) and four hour later infected with HSV.y34.5 at MO of (0.01) as shown in (A) or HSVG47Δ at MOI of (0.01) as shown in (B). Corresponding viral titers were determined 48 hours after infection from pellets. Florescence images were taken from all conditions. [n = 3; mean ± SD; *P < 0.05, ***P < 0.001, one-way analysis of variance (ANOVA), as compared to the untreated condition].

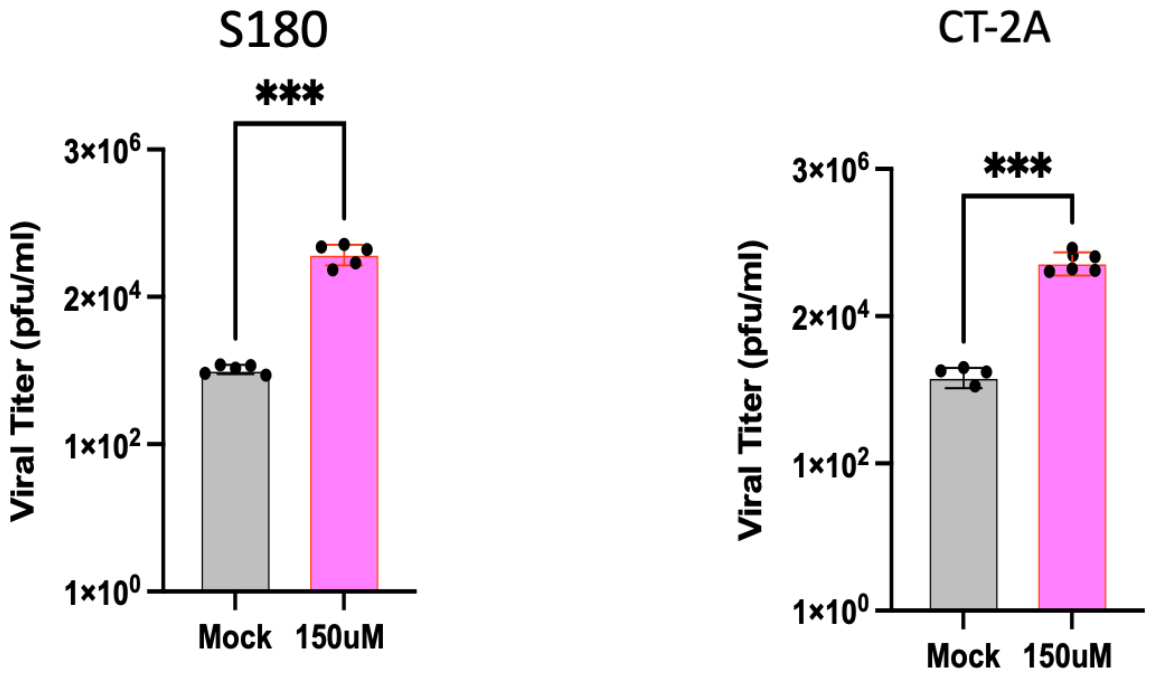
S180
***
3×10^6
2×10^4
1×10^2
1×10^0
Viral Titer (pfu/ml)
Mock
150uM
CT-2A
***
3×10^6
2×10^4
1×10^2
1×10^0
Viral Titer (pfu/ml)
Mock
150uM

Figure S4: DMF promotes HSV.n212 infection in S180 and CT2A tumour cores. Infectious viral particles were quantified from S180 and CT2A tumour cores upon treatment as described in Figure 2 by standard plaque assay.

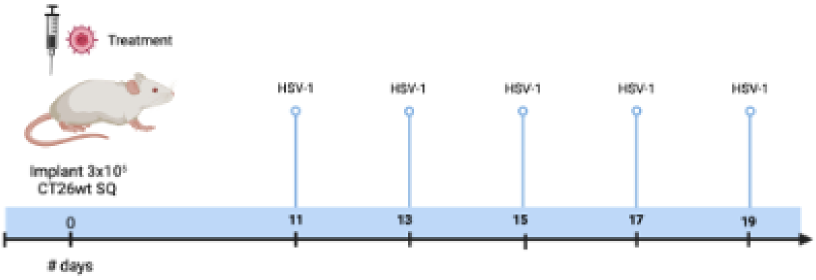
Treatment
HSV-1
HSV-1
HSV-1
HSV-1
HSV-1
Implant 3x10⁵ CT26wt SQ
0
11
13
15
17
19
days

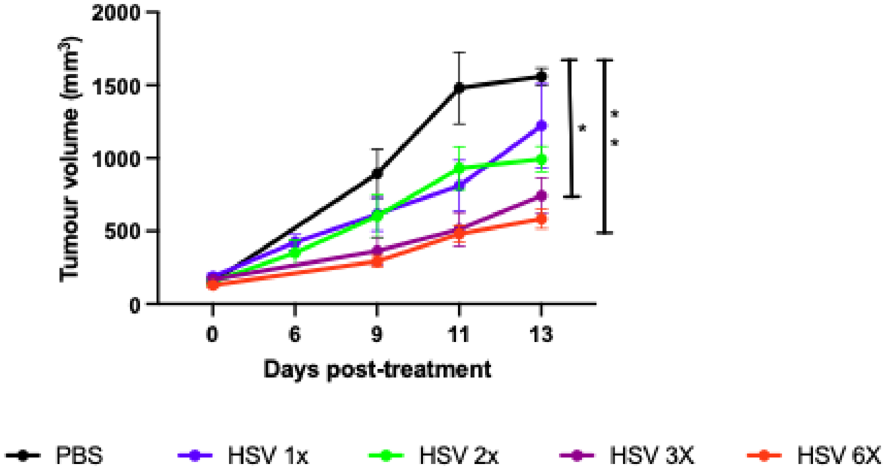
Tumour volume (mm³)
2000
1500
1000
500
0
0
6
9
11
13
Days post-treatment
*
*
*
PBS
HSV 1x
HSV 2x
HSV 3X
HSV 6X

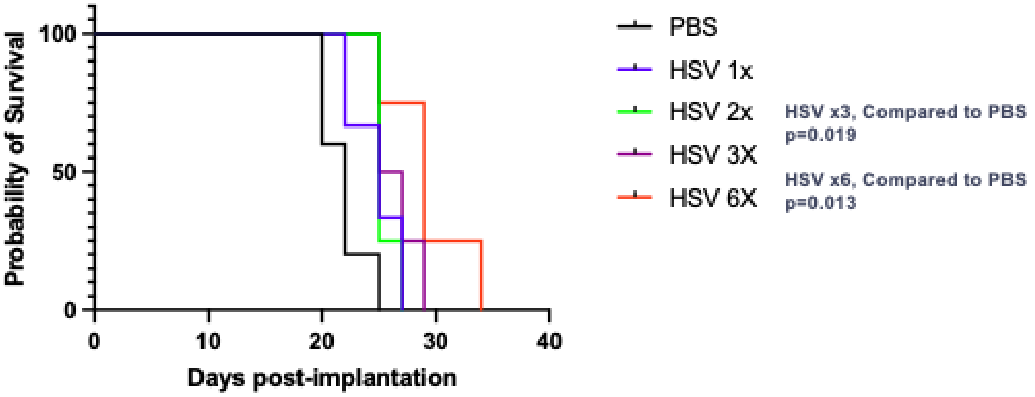
Probability of Survival
100
50
0
0
10
20
30
40
Days post-implantation
PBS
HSV 1x
HSV 2x
HSV 3X
HSV 6X
HSV x3, Compared to PBS p=0.019
HSV x6, Compared to PBS p=0.013

Figure S5: HSV.n212 dose optimization in vivo. BALB/c mice were implanted with 3×10^5 CT26.wt cells. Once tumors were ~100 mm^3, mice were treated with several doses as indicated in the diagram with the regimen of HSV.n212 (1×10^8 pfu) or PBS (intratumorally). Tumor growth and survival (endpoint tumor size of 1500 mm^3) was monitored over time.

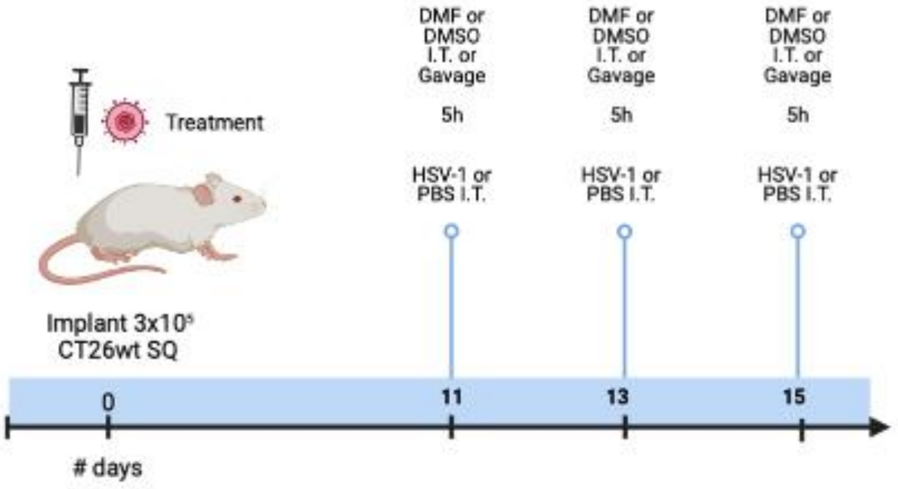
Treatment
Implant 3x10⁵
CT26wt SQ
DMF or
DMSO
I.T. or
Gavage
5h
HSV-1 or
PBS I.T.
0
11
13
15
days

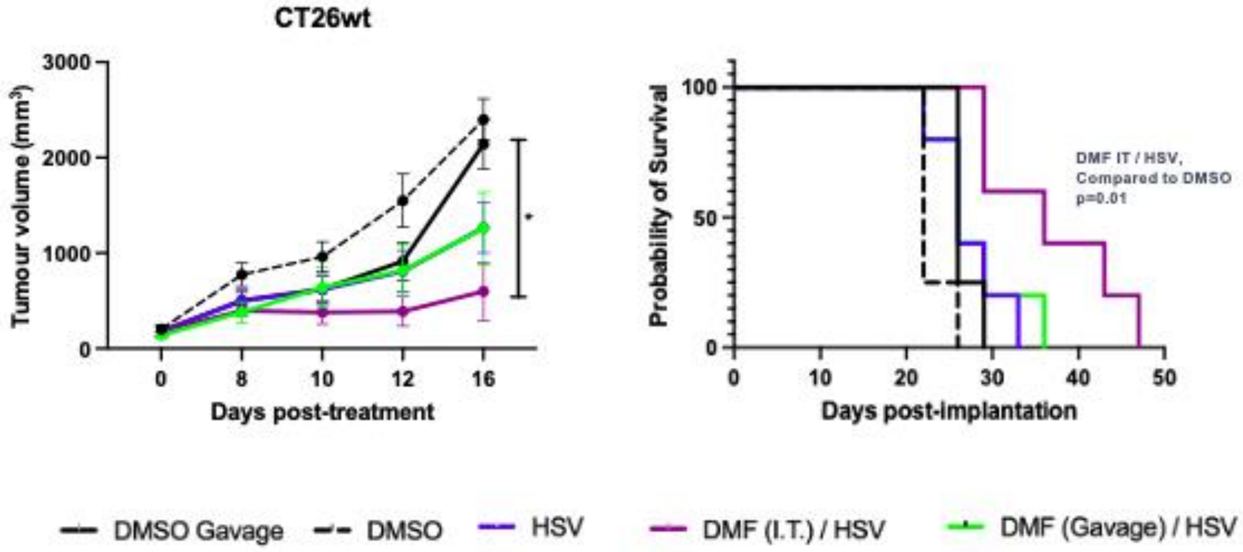
CT26wt
Tumour volume (mm³)
3000
2000
1000
0
0
8
10
12
16
Days post-treatment
*
Probability of Survival
100
50
0
0
10
20
30
40
50
Days post-implantation
DMF IT / HSV,
Compared to DMSO
p=0.01
DMSO Gavage
DMSO
HSV
DMF (I.T.) / HSV
DMF (Gavage) / HSV

Figure S6: DMF Administered intratumorally improved survival compared to gavage. Colon CT26.wt tumors were implanted with 3×10^5 into the right flank of BALB/c mice. Upon reaching ~100 mm^3, tumors were injected intratumorally with HSV.n212 or with either dimethyl sulfoxide (DMSO) or DMF (200mg/kg, either i.t. or gavage). Tumor volumes were monitored every 2-3 days. Mice were culled when tumor volumes reached 1500 mm^3 for survival analysis. Kaplan-Meier curves were plotted and compared using the log-rank (Mantel-Cox) test.
